# Genetic characterization of bovine coronavirus strain isolated in Inner Mongolia of China

**DOI:** 10.1186/s12917-024-04046-3

**Published:** 2024-05-18

**Authors:** Fan Zhang, Chunxia Chai, Rui Niu, Yun Diao, Yanyan Zhou, Jinlong Zhang, Lin Feng, Chunming Yao, Youzhi Wu, Yanhua Ma, Xiaohui Zan, Wei Wang

**Affiliations:** 1 State Key Laboratory of Reproductive Regulation and Breeding of Grassland Livestock, Inner Mongolia University, Hohhot, 010030 China; 2 Veterinary Research Institute, Inner Mongolia Academy of Agricultural & Animal Husbandry Sciences, Hohhot, 010031 China; 3 Helinger County Bureau of Agriculture and Animal Husbandry, Hohhot, 011500 China

**Keywords:** Bovine coronavirus, Genetic characterization, Isolated strain, China

## Abstract

**Background:**

Bovine coronavirus (BCoV) is implicated in severe diarrhea in calves and contributes to the bovine respiratory disease complex; it shares a close relationship with human coronavirus. Similar to other coronaviruses, remarkable variability was found in the genome and biology of the BCoV. In 2022, samples of feces were collected from a cattle farm. A virus was isolated from 7-day-old newborn calves. In this study, we present the genetic characteristics of a new BCoV isolate. The complete genomic, spike protein, and nucleocapsid protein gene sequences of the BCoV strain, along with those of other coronaviruses, were obtained from the GenBank database. Genetic analysis was conducted using MEGA7.0 and the Neighbor-Joining (NJ) method. The reference strains’ related genes were retrieved from GenBank for comparison and analysis using DNAMAN.

**Results:**

The phylogenetic tree and whole genome consistency analysis showed that it belonged to the GIIb subgroup, which is epidemic in Asia and America, and was quite similar to the Chinese strains in the same cluster. Significantly, the S gene was highly consistent with QH1 (MH810151.1) isolated from yak. This suggests that the strain may have originated from interspecies transmission involving mutations of wild strains. The N gene was conserved and showed high sequence identity with the epidemic strains in China and the USA.

**Conclusions:**

Genetic characterization suggests that the isolated strain could be a new mutant from a wild-type lineage, which is in the same cluster as most Chinese epidemic strains but on a new branch.

**Supplementary Information:**

The online version contains supplementary material available at 10.1186/s12917-024-04046-3.

## Background

Bovine coronavirus (BCoV) is linked to severe diarrhea in calves as well as the complex of respiratory diseases that affect cattle, and is also closely related to human coronavirus. Similar to other coronaviruses, BCoV exhibits significant genetic and biological variability. In 1973, Mebus et al. first reported a coronavirus that can cause severe diarrhea in calves in the United States [1]. In 1984, McNulty et al. isolated BCoV from the lung of a calf with bronchopneumonia; this strain was capable of inducing upper respiratory tract infection symptoms [2]. Subsequently, diarrhea and respiratory symptoms caused by BCoV were reported in Canada, the Netherlands, Japan, China and Ghana [2–7]. Since 2000, the most notable cases of coronavirus infection in humans have been the SARS-CoV infection in 2003 and the SARS-CoV-2 infection in 2019. Both of these coronaviruses and BCoV belong to the β-*Coronavirus* genus [8].

Coronavirus is one of the largest known RNA viruses, with a single-stranded positive RNA genome [9], and belongs to the family *Coronaviridae* in the order *Nidovirales* within the *Coronavirinae* subfamily [10]. The four main structural proteins coded by the coronavirus are the spike (S), envelope (E), membrane (M), and nucleocapsid (N) proteins [11]. S protein mediates cell adsorption and virus-membrane fusion [12], which is related to virus virulence and tissue receptor recognition, and is an important determinant of virus infection host range and cross-species infection [13, 14]. E protein is a small, major structural protein and is involved in virus assembly, budding, envelope formation and pathogenesis. its functions as an ion-channeling viroporin and interactions with both other CoV proteins and host cell proteins [15]. M protein plays an important role in virus assembly, and its interactions with the E facilitate virion production [16].N protein plays an essential role in virion assembly through its interactions with the large, positive-strand RNA viral genome and the carboxy-terminal endodomain of the M protein [17]. It is often used as a target gene for BCoV molecular characterization and molecular diagnosis [18, 19].

According to the genetic evolution analysis, BCoV may come from mutation events similar to SARS [20]. With the increase of epidemiological data, BCoV in the world is mainly divided into the GI group and GII group, according to the phylogenetic tree construction analysis based on S gene. Further analysis showed that some strains in Asia, America, Europe and early classical strains such as the original Mebus strain formed the GIa subgroups. The European BCoV strains are the most common member of the GIb subgroup. Among the GII group strains, most of the BCoV strains from the United States, China, Japan and Vietnam belong to the GIIb subgroup, while the BCoV in Korea is different from other Asian countries and belongs to the GIIa subgroup [21]. The Asian strains are closely related to the American strains and show the geographical aggregation of genetic variation. This may be related to trade between the USA and these countries in Asia [5, 22, 23]. In recent years, with the reverse globalization of trade, the virus strains prevalent in various countries mainly come from recombinant and mutant strains, and most of the isolates in the same species belong to the same lineages. There are mainly two subclusters reported in China. It comes from cow and calf subclusters, or yaks [24, 25]. The strain isolated in this study belongs to the Chinese strains cluster and is a new branch.

## Results and discussion

**Fig. 1:**
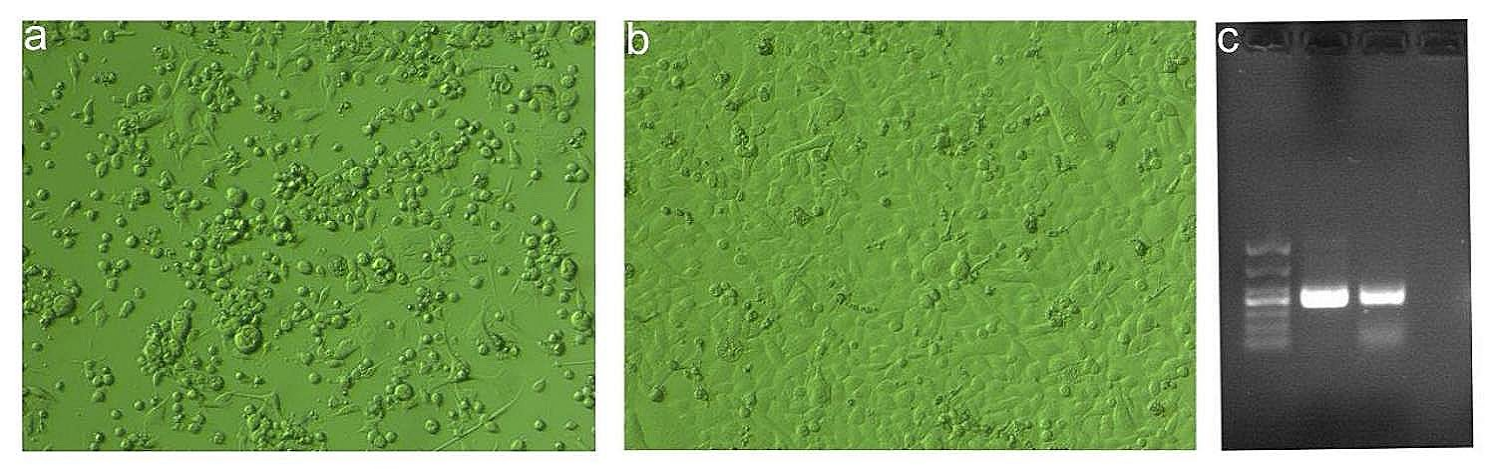
The isolation and identification of virus.(a) CPE appeared at 72 h. (b) Negative control. (c) RT-PCR identification results, Line1: Takara DL1000 DNA Marker, Line2: sample, Line3: positive control

Calf diarrhea is one of the main issues facing cattle ranches and has cost China’s cattle sector a significant amount of money. At present, according to Gene bank database, BCoV can be isolated from cow, calf, and yak in China. For example, BCoV7/2021/CHN (ON142320.1) comes from cow, SWUN/ NGG-D10/2020 (MW711287.1) from calf and YAK/HY24/CH/2017 (MH810163.1) from yak. In this study, the clinical samples were inoculated into HCT-8 cells and passaged continuously until cytopathic effects (CPE) appeared. The cells of the strain were observed under microscope at 100 times after being cultured for 72 h. The cells showed voids of different sizes, became round and pulled the net, while there was no CPE in the negative control. After the virus was collected and identified by RT-PCR, the results showed that it was positive (Fig. 1). Then the whole genome of the virus was sequenced. The sequences of the newly isolated BCoV/NMG1/2022 strains were compared with those from China, the USA, Japan and South Korea in the Genbank database. These sequences included the complete genome, S gene and N gene sequences (Table S1 in supplementary material).

**Fig. 2:**
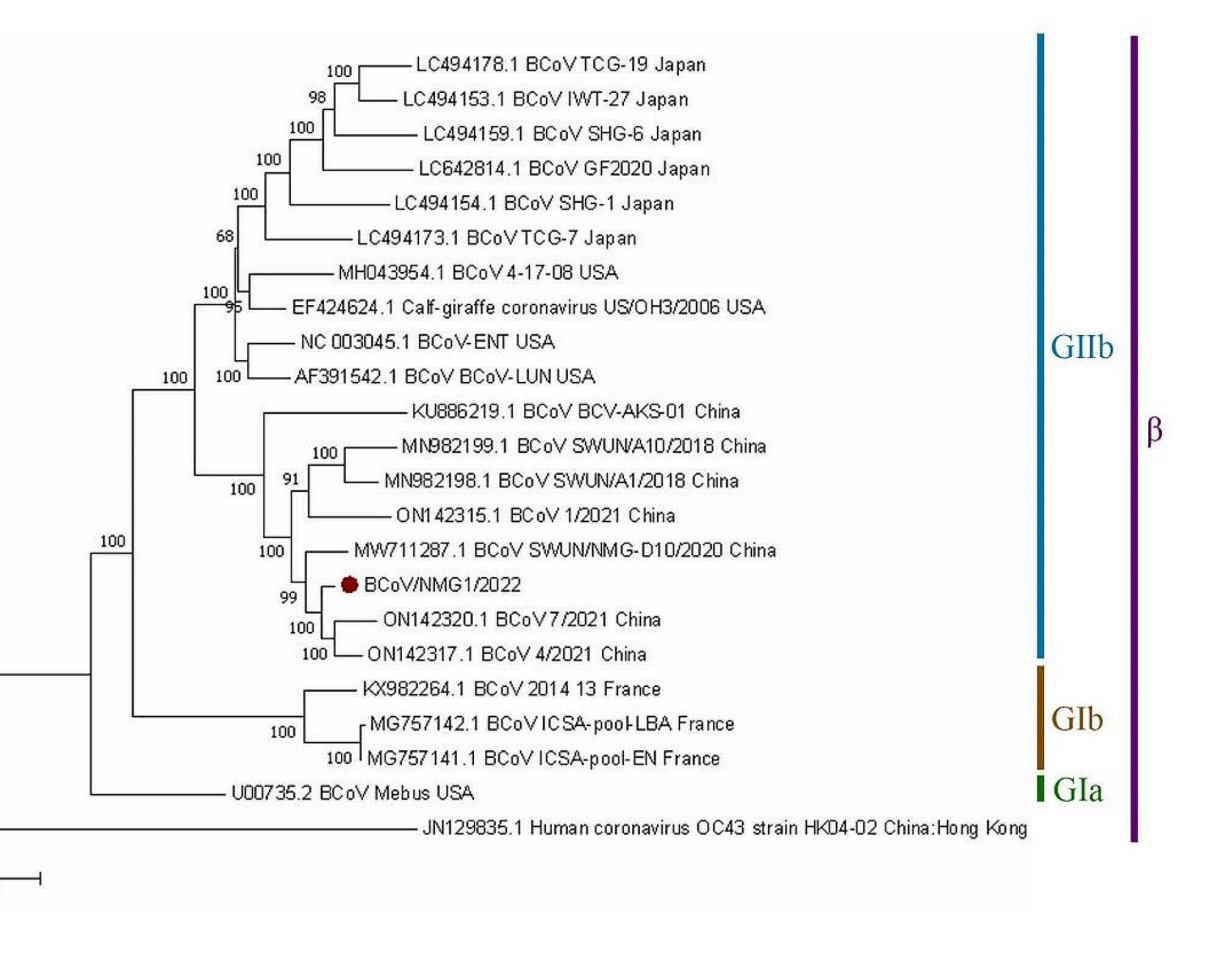
Phylogenetic trees with Neighbor-Joining show the relationship between whole genome sequence from BCoV/NMG1/2022, marked with red round, and other BCoV and coronavirus whole genome sequences from GenBank.

The isolated BCoV/NMG1/2022 strain was identified as part of the GIIb subgroup, which was primarily epidemic in Asia and America. It was very similar to the endemic strains in China in the same cluster, especially the highest consistency with 99.68% of BCoV4/2021/CHN (ON142317.1), according to a thorough genome consistency analysis and phylogenetic tree (Table S1 and Fig. 2). Calf-giraffe US/OH3/2006 (EF424624.1) isolated from giraffes had a consistency of 99.01%. The consistency of BCoV/NMG1/2022 with the original strain Mebus in the GΙ subgroup in the USA was 98.29%. The Human coronavirus OC43 strain HK04-02 (JN129835.1) from Hong Kong, China, showed a consistency of 92.04%. BCoV/NMG1/2022 was far from MERS-CoV, SARS-CoV and SRAS-CoV-2, but belonged to the same β *Coronavirus* genus. BCoV maintains a certain genetic stability [26], while BCoV has the ability to infect multiple hosts, which can be confirmed by reports of wild animals and children infected with BCoV [27–29]. According to reports, the OC43 strain may originate from the recombination of the virus transmitted by zoonosis, which may be the argument for inter specific transmission from cattle to humans [30].

**Fig. 3:**
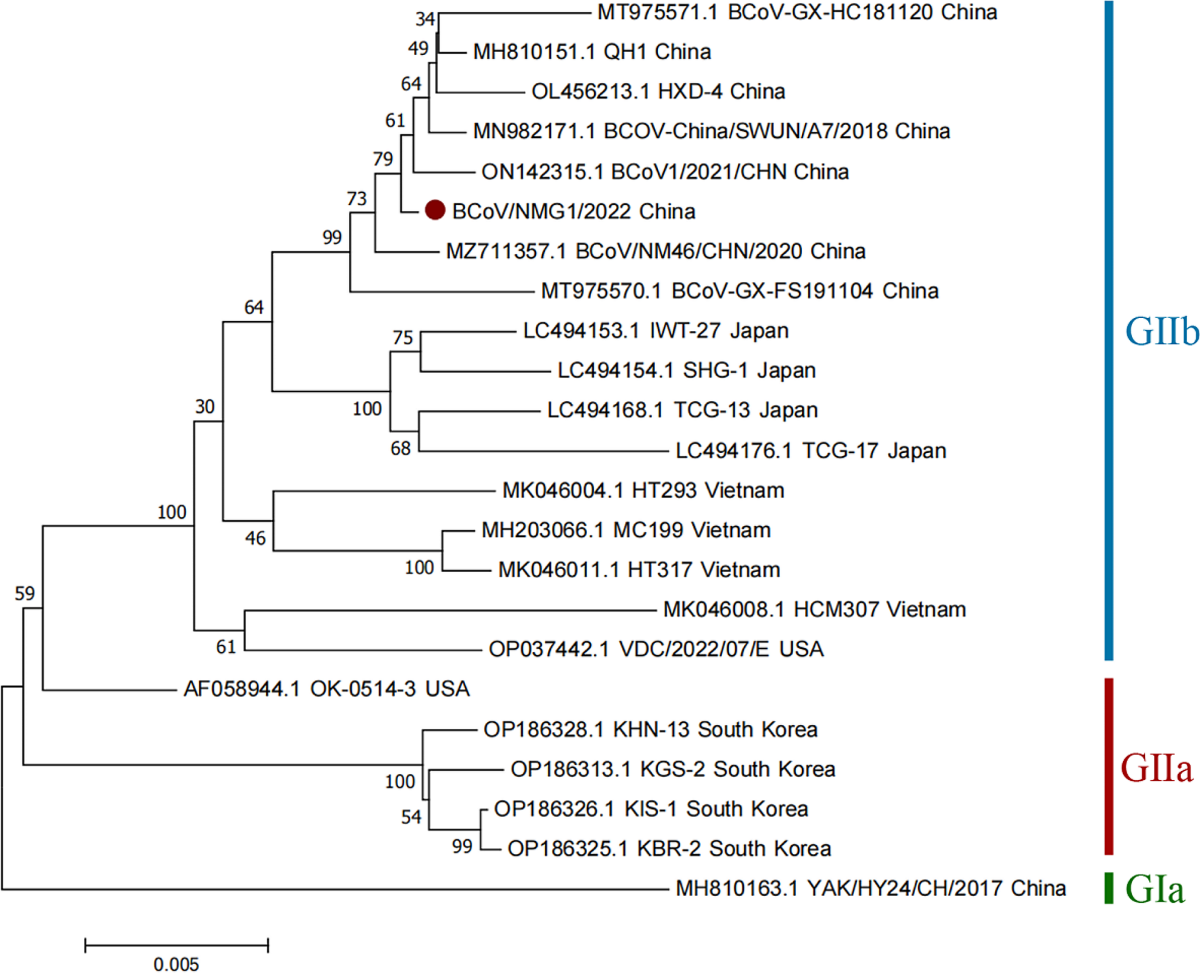
Phylogenetic trees with Neighbor-Joining show the relationship between S gene sequences from BCoV/NMG1/2022, marked with red round, and other BCoV gene sequences from GenBank.

The S gene has high mutagenicity, and the mutation site on the S gene may also be an important marker for the differentiation of the GI and GII groups [24]. According to the sequence analysis of S gene, the mutagenic property of coronavirus is related to its strong ability of genome recombination, which makes it adapt to the new host [31]. Host range expansion is associated with the recombination and mutation of the S gene [32]. Sequence alignment and phylogenetic analysis of the S gene revealed that BCoV/NMG1/2022 clusters with the predominant epidemic strains in China and differs from those in Japan and Vietnam, indicating recombination and mutation in BCoV genes across various countries and regions, leading to geographic clustering. However, the main epidemic subgroup in South Korea, which is different from Asia, is the GIIa subgroup [21].

It is worth noting that the strains with close genetic relationship are not only from cows, but also in the same cluster as the S gene QH1 (MH810151.1) from yaks, and the consistency is 99.78%. However, the YAK/HY24/CH/2017 (MH810163.1) strain isolated from yaks in China in 2017 belonged to the GIa subgroup, with 97.02% consistency with QH1 (MH810151.1) and 97.58% with BCoV/NMG1/2022 (Table S1 and Fig. 3). Recombination events were not detected in BCoV/NMG1/2022. Furthermore, twenty-three reference sequences of complete S genes from different BCOV strains were analyzed, it showed that the amino acid sequence of the S gene of the BCoV/NMG1/2022 strain has six identical amino acid mutation sites (A12T, L154F, P174S, S718L, S927A and N1192Y) with the six BCoV strains(Table S2). Due to the lack of bovine coronavirus vaccination in China, this isolate might have originated from mutating wild strains, exacerbated by China’s livestock trade.

**Fig. 4:**
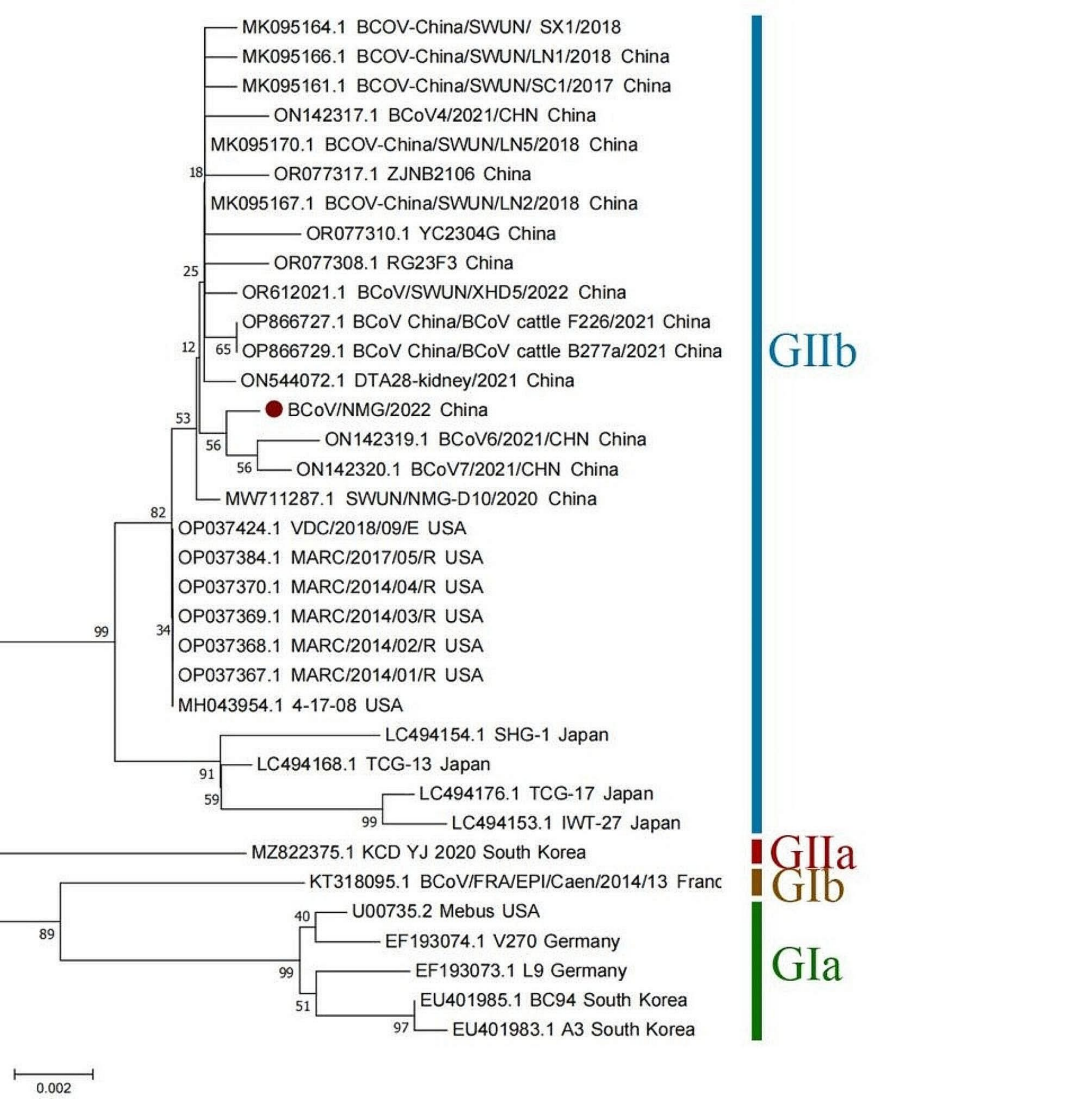
Phylogenetic trees with Neighbor-Joining show the relationship between N gene sequences from BCoV/NMG1/2022, marked with red round, and other BCoV gene sequences from GenBank.

The N gene is notably conserved. A study suggests that N gene is suitable to be used as a marker for BCoV molecular and phylogenetic analysis [33]. The N gene sequence consistency analysis and phylogenetic tree showed that the isolate BCoV/NMG1/2022 formed a new branch. The highest consistency was 99.85% with BCoV-China/SWUN/LN2/2018 (MK095167.1), 99.78% with American VDC/2018/09/E (OP037424.1) and MARC/2017/05/R (OP037384.1), 99.33% with Japanese isolate TCG-13 (LC494168.1) in 2009, 98.22% with German V270 (EF193074.1) and 98.29% with French BCoV 2014 13 (KX982264.1). The original strain Mebus exhibited 98.29% similarity (Table S1 and Fig. 4). In South Korea, a neighboring country of China, the prevalence of BCoV in diarrheal calves was very low. The presence of BCoV was significantly associated with 31–60 days of diarrhea in calves, but not in newborn calves [33]. This is different from this study, the samples in this study are from 7-day-old newborn calves, whereas in China, it is believed that BCoV usually infects 1-15-day-old calves, causing diarrhea and respiratory symptoms. This may be due to the genetic difference between the virus strains, which may lead to the difference in the age of infection. Exploring the link between these two differences could yield valuable insights.

## Conclusion

This study reports on the new BCoV isolate, BCoV/NMG1/2022. Genetic analysis indicates a high degree of similarity between the S gene of the isolate and that of yak-derived BCoVs, suggesting it is a potentially new wild-type mutation within the same cluster as many Chinese epidemic strains, albeit in a distinct branch. This strain has led to infections among newborn calves, resulting in economic losses for breeders. No vaccination strategy has been implemented in China thus far. Consequently, etiological surveillance and epidemiological investigation of susceptible animals are necessary, and it is also helpful for researchers to understand the genetic characteristics of bovine coronavirus. This information is vital for vaccine research.

## Methods

### Clinical histories and collections of samples

Samples were collected from a farm in Baotou, central Inner Mongolia. The farm encompasses an area of 5.33 million square meters of natural grassland and is home to 5,000 sheep and 2,000 cattle. It is about 150 km away from the Mongolia border. Researchers collected 11 fecal samples from 30 symptomatic calves using flocking swabs, one of which was from a 7-day-old calf. The calf was very weak and in a dying state at the time of collection and died within 1 h after the sample was collected. BCoV and BRV were detected by RT-PCR, and the detection rate was 36.4% and 27.3%, respectively, The calf had diarrhea and respiratory symptoms, with obvious pathological changes in the colon and lung. Fecal samples were collected in a sterile tube containing virus transport medium and stored at 4℃.

### Sample processing

The fecal swab (containing about 0.1 g of feces) was dissolved in 1mL RPMI1640 (Gibco, USA) containing antibiotics. The samples were homogenized with a tissue homogenizer at a frequency of 60 Hz for 10 min to prepare the suspension. The fecal homogenate underwent two freeze-thaw cycles to release the viruses, followed by centrifugation at 5,000 rpm for 10 min at 4℃ to remove any coarse particles. The supernatant from the homogenate was collected in a separate sterile tube for BCoV RT-PCR detection and virus isolation.

### Agents identification

Total RNA was extracted from samples using a QIAamp Viral RNA Kit(QIAGEN, Germany), cDNA was synthesized using a PrimeScript™ 1st Strand cDNA Synthesis Kit (Takara, Japan), following the manufacturer’s instructions. Primers reported by H. Tsunemitsu [34] were used: F-5’-GCCGATCAGTCCGACCAATC-3’; R-5’-AGAATGTCAGCCGGGGTAT-3’. The N gene was identified by targeting PCR activation at 94 ℃ for 4 min and 35 cycles of 94 ℃ for 1 min for denaturation, 1 min of primer annealing at 58℃ and 72 ℃ for 2 min for extension. The final extension was done at 72 ℃ for 7 min. The PCR assay was performed in a 25 µL volume, including 12.5 µL of Dream Taq green PCR master mix (2×) (Thermo Scientific, Germany), 1 µL of each primer (10 pmol/µL), 8.5 µL of deionized water, and 2 µL of DNA template.

### Virus isolation

Following positive PCR results, the fecal supernatant was filtered with a 0.45 μm filter and treated with 10 µg/mL trypsin (Gibco, USA) for 30 min, then the treated fecal supernatant was inoculated for 1 h. HCT-8 cells, obtained from the China Center for Type Culture Collection, were cultured at 37℃ with 5% CO_2_ in RPMI 1640 containing 5 µg/mL trypsin for 3 days after the inoculum was discarded. After observing CPE (cytopathic effect), the virus was collected.

### Nucleotide sequencing

In order to further confirm the identity of the virus, the virus genome was extracted, and the next-generation sequencing (NGS) was used to sequence the complete genome by Illumina NovaSeq6000. First, the ABySS (http://www.bcgsc.ca/platform/bioinfo/software/abyss) software, which is a de novo sequence assembler, was employed to assemble the sequence data using multiple k-mer parameters to achieve the best assembly result. Subsequently, GapCloser software (https://sourceforge.net/projects/soapdenovo2/files/GapCloser/) was used to perform gap filling and base correction of the assembly, improving the local continuity and accuracy of the sequence. The complete genome sequence was submitted to the Genbank database with accession numbers of OP924545.1 (Bovine coronavirus isolate strain BCoV/NMG1/2022, complete genome).

### Phylogenetic analysis

Sequence alignment was performed using the GenBank database, from which the whole genome sequence and the S and N gene sequences of BCoV were downloaded. These sequences were compared and analyzed by MEGA7.0 and ClustalW, the phylogenetic tree was constructed using the Neighbor-Joining (NJ) method. The bootstrap value was set to 1000. DNAMAN software was used to compare and analyze the related genes of reference strains collected from GenBank. Recombination events were assessed using RDP 4 software with the RDP, GeneConv, Chimaera, MaxChi, BootScan, SiScan, and 3Seq methods. A total of twenty-three reference sequences of complete S genes from different BCoV strains were analyzed by MEGA7.0.

### Electronic supplementary material

Below is the link to the electronic supplementary material.

Supplementary Material 1

Supplementary Material 2

## Data Availability

The data that support the findings of this study are available from the corresponding author upon reasonable request. The datasets generated and/or analyzed during the current study are available in the GenBank database.
