# Supplementary material for: Genetic characterization of bovine coronavirus strain isolated in Inner Mongolia of China
Source: BMC Vet Res. 2024 May 18;20:209. doi: 10.1186/s12917-024-04046-3 (PMC11102244; doi:10.1186/s12917-024-04046-3)
Supplement: Supplementary file 1 — Supplementary Material 1 [file 12917_2024_4046_MOESM1_ESM.docx]

**Table S1** The nucleotide consistency between BCoV/NMG1/2022 and other Bovine coronavirus gene sequences.

|  | Genbank ID | Abbreviationname | Virus type | Year | Origin | Consistency with BCoV/NMG1/2022 | | |
| --- | --- | --- | --- | --- | --- | --- | --- | --- |
|  |  |  |  |  |  | complete genome | S | N |
| 1 | ON142317.1 | BCoV4/2021/CHN | BCoV | 2021 | China | 99.68 | 99.68 | 99.70 |
| 2 | ON142320.1 | BCoV7/2021/CHN | BCoV | 2021 | China | 99.67 | 99.44 | 99.78 |
| 3 | MW711287.1 | SWUN/NMG-D10/2020 | BCoV | 2020 | China | 99.59 | 99.54 | 99.78 |
| 4 | ON142315.1 | BCoV1/2021/CHN | BCoV | 2021 | China | 99.46 | 99.76 | 99.48 |
| 5 | MN982199.1 | BCoV-China/SWUN/A10/2018 | BCoV | 2018 | China | 99.29 | 99.24 | 99.33 |
| 6 | MN982198.1 | BCoV-China/SWUN/A1/2018 | BCoV | 2018 | China | 99.39 | 99.41 | 99.33 |
| 7 | KU886219.1 | BCV-AKS-01 | BCoV | 2015 | China:Xinjing | 99.10 | 98.80 | 97.92 |
| 8 | MN982171.1 | BCoV-China/SWUN/A7/2018 | BCoV | 2018 | China | / | 99.78 | / |
| 9 | MZ711357.1 | BCoV/NM46/CHN/2020 | BCoV | 2020 | China | / | 99.73 | / |
| 10 | OL456213.1 | HXD-4 | BCoV | 2021 | China | / | 99.61 | / |
| 11 | MT975570.1 | BCoV-GX-FS191104 | BCoV | 2019 | China | / | 99.32 | / |
| 12 | MT975571.1/MK757493.1 | BCoV-GX-HC181120 | BCoV | 2018 | China | / | 99.44 | / |
| 13 | MH810151.1/MH741383.1 | QH1 | Yak coronavirus | 2017 | China | / | 99.78 | / |
| 14 | MH810163.1 | YAK/HY24/CH/2017 | Yak coronavirus | 2017 | China | 97.58 | 97.14 | 99.63 |
| 15 | MK095177.1/MK095164.1 | BCOV-China/SWUN/ SX1/2018 | BCoV | 2018 | China | / | 99.17 | 99.70 |
| 16 | MK095174.1/MK095161.1 | BCOV-China/SWUN/SC1/2017 | BCoV | 2017 | China | / | 99.12 | 99.70 |
| 17 | MK095180.1/MK095167.1 | BCOV-China/SWUN/LN2/2018 | BCoV | 2018 | China | / | 98.90 | 99.85 |
| 18 | MK095183.1/MK095170.1 | BCOV-China/SWUN/LN5/2018 | BCoV | 2018 | China | / | 99.44 | 99.78 |
| 19 | LC494178.1 | TCG-19 | BCoV | 2016 | Japan | 98.51 | 98.58 | 98.66 |
| 20 | LC494176.1 | TCG-17 | BCoV | 2016 | Japan | 98.50 | 98.36 | 98.96 |
| 21 | LC494168.1 | TCG-13 | BCoV | 2009 | Japan | 98.70 | 98.83 | 99.33 |
| 22 | LC494153.1 | IWT-27 | BCoV | 2017 | Japan | 98.51 | 98.92 | 98.59 |
| 23 | LC494159.1 | SHG-6 | BCoV | 2017 | Japan | 98.44 | 98.66 | 98.81 |
| 24 | LC642814.1 | GF2020 | BCoV | 2020 | Japan | 98.48 | 98.70 | 99.26 |
| 25 | LC494154.1 | SHG-1 | BCoV | 2014 | Japan | 98.53 | 98.80 | 98.37 |
| 26 | LC494173.1 | TCG-7 | BCoV | 2008 | Japan | 98.72 | 99.00 | 99.11 |
| 27 | NC_003045.1 | BCov-ENT | BCoV | 2001 | USA | 98.94 | 98.90 | / |
| 28 | AF391542.1 | BCov-LUN | BCoV | 2001 | USA | 98.91 | 98.70 | 99.41 |
| 29 | MH043954.1 | 4-17-08 | BCoV | 2017 | USA:Pennsylvania | 98.81 | 98.90 | 99.70 |
| 30 | U00735.2 | Mebus | BCoV | 2003 | USA | 98.29 | 97.61 | 98.29 |
| 31 | EF424624.1 | US/OH3/2006 | Calf-giraffe coronavirus | 2006 | USA:Ohio | 99.01 | 98.73 | 99.70 |
| 32 | OP037442.1 | VDC/2022/07/E | BCoV | 2022 | USA:Oregon | 98.57 | 98.53 | 99.48 |
| 33 | OP037424.1 | VDC/2018/09/E | BCoV | 2018 | USA: Nebraska | 98.77 | 98.92 | 99.78 |
| 34 | AF058944.1 | OK-0514-3 | BCoV | 1998 | USA | / | 96.44 | 92.97 |
| 35 | OP037384.1 | MARC/2017/05/R | BCoV | 2017 | USA: Nebraska | 98.74 | 98.88 | 99.78 |
| 36 | MK046004.1 | HT293 | BCoV | 2018 | Vietnam | / | 98.78 | / |
| 37 | MK046008.1 | HCM307 | BCoV | 2018 | Vietnam | / | 98.12 | / |
| 38 | MK046011.1 | HT317 | BCoV | 2018 | Vietnam | / | 98.68 | / |
| 39 | MH203066.1 | MC199 | BCoV | 2017 | Vietnam | / | 98.75 | / |
| 40 | OP186325.1 | KBR-2 | BCoV | 2017 | South Korea | / | 97.58 | / |
| 41 | OP186313.1 | KGS-2 | BCoV | 2017 | South Korea | / | 97.58 | / |
| 42 | OP186328.1 | KHN-13 | BCoV | 2018 | South Korea | / | 97.63 | / |
| 43 | OP186326.1 | KIS-1 | BCoV | 2017 | South Korea | / | 97.65 | / |
| 44 | EU401987.1/EU401983.1 | A3 | BCoV | 2008 | South Korea | / | 97.58 | 98.07 |
| 45 | MW881221.1/MZ822375.1 | KCD YJ 2020 | BCoV | 2020 | South Korea | / | 96.95 | 90.79 |
| 46 | EU401989.1/EU401985.1 | BC94 | BCoV | 2008 | South Korea | / | 97.58 | 98.22 |
| 47 | KX982264.1 | BCoV 2014 13 | BCoV | 2014 | France | 97.76 | 97.24 | 98.29 |
| 48 | MG757142.1 | ICSA-pool-LBA | BCoV | 2014 | France | 97.80 | 97.24 | 98.22 |
| 49 | MG757141.1 | ICSA-pool-EN | BCoV | 2014 | France | 98.11 | 97.22 | 98.22 |
| 50 | KT318123.1/KT318095.1 | BCoV/FRA/EPI/Caen/2014/13 | BCoV | 2014 | France | / | 97.24 | 98.29 |
| 51 | EF193073.1 | L9 | BCoV | 2006 | Germany | / | / | 98.14 |
| 52 | EF193075.1/EF193074.1 | V270 | BCoV | 2006 | Germany | / | 97.68 | 98.22 |

/:no sequence in Genbank.
