# Supplementary material for: Genetic characterization of bovine coronavirus strain isolated in Inner Mongolia of China
Source: BMC Vet Res. 2024 May 18;20:209. doi: 10.1186/s12917-024-04046-3 (PMC11102244; doi:10.1186/s12917-024-04046-3)
Supplement: Supplementary file 2 — Supplementary Material 2 [file 12917_2024_4046_MOESM2_ESM.docx]

**Table S2** Twenty-three reference sequences of complete S genes and six amino acid mutation sites.

|  | Genbank ID | Abbreviationname | Year | Origin | amino acid mutation sites | | | | | |
| --- | --- | --- | --- | --- | --- | --- | --- | --- | --- | --- |
| 1 | OP924545.1 | BCoV/NMG1/2022 | 2022 | China | T | F | S | L | S | Y |
| 2 | OL456213.1 | HXD-4 | 2021 | China | T | F | S | L | S | Y |
| 3 | MT975570.1 | BCoV-GX-FS191104 | 2021 | China | A | F | S | L | S | Y |
| 4 | MT975571.1 | BCoV-GX-HC181120 | 2020 | China | T | F | S | L | S | Y |
| 5 | MT975572.1 | BCoV-GX-NN190313 | 2021 | China | A | L | P | S | A | N |
| 6 | MH203066.1 | MC199 | 2018 | China | A | F | S | S | S | N |
| 7 | MK046011.1 | HT317 | 2018 | China | A | F | S | S | S | N |
| 8 | MK046004.1 | HT293 | 2015 | China:Xinjing | A | F | S | S | S | N |
| 9 | MK046008.1 | HCM307 | 2018 | China | A | F | S | L | F | Y |
| 10 | AF058944.1 | OK-0514-3 | 2020 | China | A | L | P | S | S | N |
| 11 | OP037442.1 | VDC/2022/07/E | 2021 | China | A | F | S | L | S | N |
| 12 | OP186326.1 | KIS-1 | 2019 | China | A | L | P | S | S | N |
| 13 | OP186328.1 | KHN-13 | 2018 | China | A | L | P | S | S | N |
| 14 | OP186313.1 | KGS-2 | 2017 | China | A | L | P | S | S | N |
| 15 | OP186325.1 | KBR-2 | 2017 | China | A | L | P | S | S | N |
| 16 | LC494153.1 | IWT-27 | 2018 | China | A | F | P | S | S | N |
| 17 | LC494154.1 | SHG-1 | 2017 | China | A | F | P | S | S | N |
| 18 | LC494168.1 | TCG-13 | 2009 | Japan | A | F | P | S | S | N |
| 19 | LC494176.1 | TCG-17 | 2016 | Japan | A | F | P | S | S | N |
| 20 | MH810151.1 | QH1 | 2016 | Japan | T | F | S | L | S | Y |
| 21 | MH810163.1 | YAK/HY24/CH/2017 | 2016 | Japan | A | V | P | S | S | N |
| 22 | MN982171.1 | BCOV-China/SWUN/A7/2018 | 2009 | Japan | T | F | S | L | S | Y |
| 23 | MZ711357.1 | BCoV/NM46/CHN/2020 | 2017 | Japan | T | F | S | L | S | Y |
| 24 | ON142315.1 | BCoV1/2021/CHN | 2017 | Japan | T | F | S | L | S | Y |

Six sequences with identical amino acid mutation sites were highlighted in yellow.
